# A possible structural correlate of learning performance on a colour discrimination task in the brain of the bumblebee

**DOI:** 10.1098/rspb.2017.1323

**Published:** 2017-10-04

**Authors:** Li Li, HaDi MaBouDi, Michaela Egertová, Maurice R. Elphick, Lars Chittka, Clint J. Perry

**Affiliations:** School of Biological and Chemical Sciences, Queen Mary University of London, London E1 4NS, UK

**Keywords:** bumblebee, inter-individual learning differences, microglomeruli, mushroom bodies, synaptic plasticity, visual learning

## Abstract

Synaptic plasticity is considered to be a basis for learning and memory. However, the relationship between synaptic arrangements and individual differences in learning and memory is poorly understood. Here, we explored how the density of microglomeruli (synaptic complexes) within specific regions of the bumblebee (*Bombus terrestris*) brain relates to both visual learning and inter-individual differences in learning and memory performance on a visual discrimination task. Using whole-brain immunolabelling, we measured the density of microglomeruli in the collar region (visual association areas) of the mushroom bodies of the bumblebee brain. We found that bumblebees which made fewer errors during training in a visual discrimination task had higher microglomerular density. Similarly, bumblebees that had better retention of the learned colour-reward associations two days after training had higher microglomerular density. Further experiments indicated experience-dependent changes in neural circuitry: learning a colour-reward contingency with 10 colours (but not two colours) does result, and exposure to many different colours may result, in changes to microglomerular density in the collar region of the mushroom bodies. These results reveal the varying roles that visual experience, visual learning and foraging activity have on neural structure. Although our study does not provide a causal link between microglomerular density and performance, the observed positive correlations provide new insights for future studies into how neural structure may relate to inter-individual differences in learning and memory.

## 1. Introduction

The search for the biological basis of individual learning and memory abilities is as old as the field of cognitive science [1]. Some studies in comparative cognition work suggest a positive correlation between general cognitive ability (intelligence) and brain size [2,3]. However, the correlations found yield some inconsistencies [4] and the overall size of nervous tissue tells us little about why there are individual cognitive differences. A mechanistic understanding of individual learning ability requires examining the underlying structures within the brain and how they change in relation to learning performance. Synaptic plasticity is considered to be a basis for learning and memory [5–7]. However, how synaptic structures relate to individual learning differences is not known [8].

**Figure 1.**
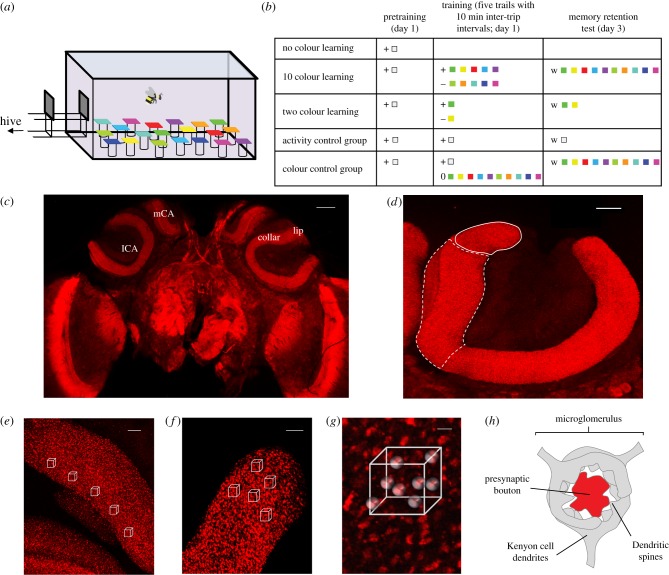
Behavioural paradigm and quantification of microglomeruli. (*a,b*) Experimental set-up and procedure. (*a*) Schematic of training/testing arena. (*b*) Training procedures for bees in each experiment (see also Material and methods). Grey squares, transparent chips; coloured squares, coloured chips (see the electronic supplementary material, figure S1); +, sucrose solution; −, quinine solution; w, water; (*c*) confocal section of the frontal view of an adult bumblebee's whole-brain immunuolabelled with anti-synapsin (scale bar, 150 µm; lCA, lateral calyx; mCA, medial calyx). (*d*) Right lateral calyx (scale bar, 50 µm; white solid line indicates lip region and white dotted line indicates collar region). (*e*) Collar and (*f*) lip regions of a mushroom body calyx (scale bar, 20 µm), individual microglomeruli can be seen labelled with anti-synapsin. White outlines are example positions of selection cuboids (white). (*g*) Enlarged immunolabelling view of an example cube. Each projection neuron bouton was visualized by spheres (shown in grey here) showing the position of each in a 7.8 µm × 7.8 µm × 7.8 µm cube (scale bar, 2 µm). (*h*) Diagram of a microglomerular complex, including a presynaptic bouton from a projection neuron (red) and the postsynaptic endings of Kenyon cell neurons (grey).

Bumblebees' and honeybees’ impressive cognitive abilities, simple neural architecture compared with mammals, and variation in individual learning performance have provided an ideal model for investigating the neural bases of memory [9–12]. The mushroom bodies of insect brains are high-level sensory integration centres that are involved in learning and memory [13,14]. The main input regions within the mushroom bodies, the calyces, comprise the *lip*, receiving olfactory information from the antennal lobe, the *collar* receiving visual information from the optic lobe, and the *basal ring*, receiving both visual and olfactory information [15–18]. Within each of these regions, the neuronal connections are organized in synaptic complexes called microglomeruli (figure 1*h*), each consisting of a single presynaptic bouton from the axon terminal of a projection neuron surrounded by several postsynaptic dendrites of intrinsic neurons, called Kenyon cells [19,20].

Identification of microglomeruli via immunolabelling has shown that age, age-dependent behavioural changes, temperature during pre-adult development, developmental changes and foraging activities of various social Hymenoptera lead to synaptic organizational and structural changes within the mushroom body calyces [8,21–24]. Recent work also showed that establishment of long-term memory is accompanied by changes in microglomerular density within the mushroom body of the insect brain, but this has only been examined for olfactory learning [25,26]. The few studies exploring how visual information affects microglomeruli in the insect brain have been limited to light exposure or deprivation [21,24,27], or a simple two-colour discrimination task (see [28,29] and Discussion). Understanding how individual learning abilities might relate to neural architecture will provide valuable information on the neural underpinnings of cognition in general. In the present study, we asked how learning several colours (a 10-colour visual discrimination task) affects the density of microglomeruli in the bumblebee brain, and whether microglomerular density correlates with individual performance during the task.

## 2. Material and methods

### (a) Animals

Bumblebee colonies (*Bombus terrestris*) were purchased from Biobest Belgium NV (Westerlo, Belgium). All colonies were settled in wooden nest-boxes (40 × 28 × 11 cm), which were connected to small flight arenas (65 × 45 × 25 cm) with a Perspex corridor (25 × 3.5 × 3.5 cm). Small doors in the corridor allowed us to control which bees were able to enter the arena at any one time. Each day, newly emerged bees were placed in a queen marking cage (EH Thorne Ltd, Market Rasen, UK) and marked with a number tag (Opalithplättchen, Warnholz & Bienenvoigt, Ellerau, Germany) superglued to the top of the thorax to identify bees individually and to know their age. Only tagged bees were used for experiments. Bees had no foraging experience prior to pretraining; bumblebees were kept within the hive nest box without access to the arena until they were trained according to the experiment. All bees used in experiments were similar in age (12.8 ± 0.2 days) at time of collection, i.e. end of experiment.

### (b) Pretraining

All bees, except those in the No-colour Learning group in experiment 3, were first trained to land on colourless transparent (Perspex) chips (25 × 25 mm) with 7 µl 40% sucrose solution, which they would consume. Chips were arranged in a pseudorandom array of 10 chips within the arena, each on top of a small glass vial. Bees successfully foraging from the transparent chips and returning to the colony eight to 10 times on a regular basis (inter-trip interval within 5 min) were moved on to the training phase (figure 1*a*,*b*). Bees foraged together for most of their trips during pretraining (a trip is defined as the time a bee spent foraging in the arena before returning to the nest to unload her collected crop).

To control for any differences in microglomerular density that may be affected by bee size differences, worker (female) bumblebees of the same size were selected visually, and later confirmed by measuring head width (maximal distance between the distal surfaces of the eyes measured in dorsal aspect), as a proxy of body size, because head width is correlated with both body size and brain volume (e.g. [30,31]). All bees used in our study had head widths from 4.2–5.1 mm—a narrow range compared with the total species range of 2.8–5.3 mm [32]. Once emptied by a bee, chips were refilled by the experimenter during pretraining and training.

### (c) Training

#### (i) Experiment 1

Bees were trained individually to discriminate five different chips (artificial flowers; coloured Perspex chips, 25 × 25 mm) containing sucrose solution from five different chips containing bitter quinine solution (figure 1*a,b*). Details on illumination and colour stimuli can be found in the electronic supplementary material, S1. In all experiments, chips were cleaned with 70% ethanol in water between two sequential trips to ensure no scent marks were being used to solve the task. Bees underwent five foraging trips with 10 min inter-trip intervals (a paradigm that has been shown to cause long-term memory formation in bees [33]). Inter-trip intervals were kept consistent. Bees tended to return from their nest to the corridor every few minutes, and when this occurred, bees were prevented from entering the arena using small doors in the corridor until the 10 min interval had ended. There were two chips of each colour and 20 chips in total in the arena. All rewarding chips contained 7 µl 40% sucrose solution, and all unrewarding chips contained 7 µl saturated quinine solution (1.2 mg ml^−1^ H_2_O). Colour loci nearest each other were split between rewarding and unrewarding (electronic supplementary material, figure S1*a*), so that the task would be more difficult. During the last 10 trials of training, bees landed more on all rewarding colours than any unrewarding colours (generalized linear mixed model (GLMM): *p* < 0.0001; electronic supplementary material, table S6), but there was no difference in landing proportions amongst rewarding colours and no difference in landing proportions among unrewarding colours. These results show that bees had no preference between any of the rewarding colours. Bees naturally returned to their nest to unload the collected sucrose solution once they filled their crop. Bees were confined to the nest for 2 days after training to prevent any further foraging experience. Bees still had access to food, because each evening the colony was fed with 40% sucrose solution pipetted directly into their honey pots every day (approx. 10 ml). On day 3, bees received a memory retention test on the same chip setting as in training, except that each chip contained 7 µl water (without sucrose). All landings on chips within 3 min of entering the arena were recorded. Age-matched bees (*n* = 30; 12.8 ± 0.4 days at end of experiment) were collected immediately after the retention test (three colonies) for immunolabelling.

#### (ii) Experiment 2

Training was performed exactly as in experiment 1 (figure 1*a*,*b*; one colony), but age-matched bees (*n* = 10; all 12 days at end of experiment) were collected immediately after the final trip of the training on the first day for immunolabelling. It has been shown that hours are required, after stimulation, for new synapses to be formed, and for synapsin to increase to levels where microglomerular complexes are visible through immunolabelling [34–36]. We collected bees immediately after training (less than 50 min) to help eliminate the possibility that any new synapses would be formed in the bumblebee brain.

#### (iii) Experiment 3

Age-matched bees (*n* = 42; 13.1 ± 0.3 days at end of experiment) were randomly assigned to three different groups. Bees in the No-colour Learning group (no pretraining) were allowed to land and feed from one clear chip, then collected immediately upon landing on a second clear chip. This collection method was employed to confirm that bees were foragers and allowed us to ensure that no long-term memory of visual information could be formed and no synaptic changes would occur within the brain areas to be examined because time of first landing to collection was less than 1 min. For the Two-colour Learning group, bees foraged on 20 chips, half of them green and with 7 µl sucrose solution, and the other half yellow, with 7 µl quinine solution. The spectral reflectance of green and yellow is shown in the electronic supplementary material, figure S1*a*–*c*. Each bee was trained individually and had five foraging trips with an inter-trip interval of 10 min. The 10-colour Learning group experienced the same training procedure as bees in experiment 1 (figure 1*b*). All bees in the Two-colour and 10-colour Learning groups were collected immediately after the memory retention test conducted 2 days after training for immunolabelling. Comparing these groups gave us three levels of learning (no colour, some colour and several colours) to compare with measured microglomerular density in the visual input region of the bee mushroom bodies.

#### (iv) Experiment 4

Age-matched bees (*n* = 37; 12.6 ± 0.3 days at end of experiment) were randomly assigned to three different groups. The 10-colour Learning group experienced the same training procedure as in experiment 3. Bees in the Activity Control group were trained to associate 20 clear chips with reward, so that they received the same foraging experience as the 10-colour Learning group, but without experiencing any colours. Bees in the Colour Control group were trained to associate five clear chips with reward while 20 coloured chips were in the arena at the same time, so that they received the same foraging experience and colours as the 10-colour Learning group, but without learning to discriminate any colours. These 20 coloured chips contained no reward or water, and bees did not ever land on these coloured chips, and therefore we know that bees did not learn any rewarding or punishing association with the colours. All three groups of bees received the same training protocol, five foraging trips with 10 min inter-trip intervals. All bees were trained individually and collected immediately after the memory retention test conducted 2 days after training for immunolabelling.

### (d) Quantification of microglomeruli in the mushroom body calyces

We established a methodology for immunolabelling of presynaptic terminals in whole-mount brains that enabled identification of microglomeruli, employing an antibody to the synaptic vesicle-associated protein synapsin I. Our method combined the procedures from two previous studies [23,37] and the detailed procedures can be found in the electronic supplementary material, S2.

### (e) Statistical analysis

Statistical tests were conducted with Matlab (MathWorks, Natick, MA, USA). Details on statistical analyses can be found in the electronic supplementary material, S3.

## 3. Results

### (a) Memory retention and learning speed correlate with microglomerular density

The two-colour visual discrimination task is a classic training paradigm for studying bee learning and memory [38–40]. However, this is an easy task where performance variation between individuals is limited when colours are easily distinguishable; learning speed is consistently fast and memory retention is reliably good across individual bees given similar training. We thus designed a more sensitive (to individual differences) 10-colour Learning paradigm, where bees had to distinguish five different rewarding colours from five different punishing colours (figure 1*a,b*). The rationale for this design was to make the visual task difficult enough to quantify differences in learning performance across individuals. Bumblebees took more trips to remember all five rewarding colours and avoid all five unrewarding colours, compared with Two-colour Learning (*t*-test; *t*_25_ = −4.61, *p* = 1.02 × 10^−4^), and individual differences in memory retention varied enough to be examined (two-colour task: 93–100% choice accuracy; s.d. = 2%; 10-colour task = 73–100% choice accuracy; s.d. = 10%).

**Figure 2.**
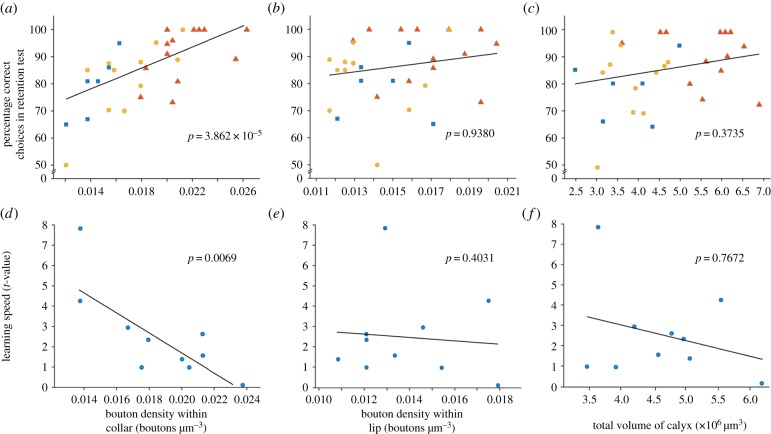
The relationship between microglomerular density and behaviour. (*a*) Microglomerular density in the collar correlated significantly and positively with memory retention in experiment 1 (GLMM: *p* < 0.0001; electronic supplementary material, table S1). No correlation was found in the lip (*b*) or calyx volume (*c*). (*a*–*c*) Symbols indicate different colonies (*n* = 31 bees; colonies 1–3); experiment 1. (*d*) There was a significant positive correlation between microglomerular density in the collar and learning speed (number of errors during training) in experiment 2 (GLMM: *p* = 0.0069; electronic supplementary material, table S2). Again, no correlation was found in the lip (*e*) or calyx volume (*f*). (*d–f*), *n* = 10 bees; experiment 2. The *t*-value was the indicator for learning speed (Material and methods). High *t*-values indicate slow learning whereas low *t*-values indicate fast learning. Generalized linear mixed model (GLMM) analysis was conducted and the significance *p*-value of each factor is shown in each panel. Solid lines are lines of best fit on the means obtained from the generalized mixed model (electronic supplementary material, tables S1 and S2). (Online version in colour.)

In experiment 1, bees (*n* = 30) learned to land on five rewarding chips and to not land on five unrewarding chips (Material and methods; figure 1*a*,*b*). Two days after training, bees received a memory retention test on the same set-up as in training. Bees were collected immediately after the retention test to examine microglomerular density in the mushroom bodies. Only the density of microglomeruli in the collar region of the mushroom body calyx was significantly, and positively, correlated with memory retention (GLMM: *p* < 0.0001, figure 2*a*; electronic supplementary material, table S1). Microglomerular density in the lip region did not correlate with long-term memory retention, nor did calyx volume (figure 2*b*,*c*; electronic supplementary material, table S1).

It could be that better memory performance in experiment 1 resulted from better learning. Each bee learned the task to proficiency (100% performance on the last trip), and so this could not be a determining factor. However, bees did reach proficiency at different rates, i.e. bees' learning speeds varied. Therefore, to explore whether bees’ faster speed of learning causes higher microglomerular density in the collar region and this then results in better memory performance, or whether it is already in place to allow for better performance, we trained another group of bees (*n* = 10) on the same visual learning paradigm as experiment 1 above, but each bee was collected immediately after training (experiment 2, Material and methods). As with memory performance, only microglomerular density in the collar correlated significantly, and positively, with learning speed (GLMM: *p* = 0.0069, figure 2*d*; electronic supplementary material, table S2). Microglomerular density in the lip region did not correlate with learning speed, nor did calyx volume (figure 2*e*,*f*; electronic supplementary material, table S2). These findings suggest that a higher microglomerular density within the mushroom body collar may predispose bees to better performance in both learning speed and long-term memory retention in a visual learning task.

### (b) Visual learning correlates with greater microglomerular density

**Figure 3.**
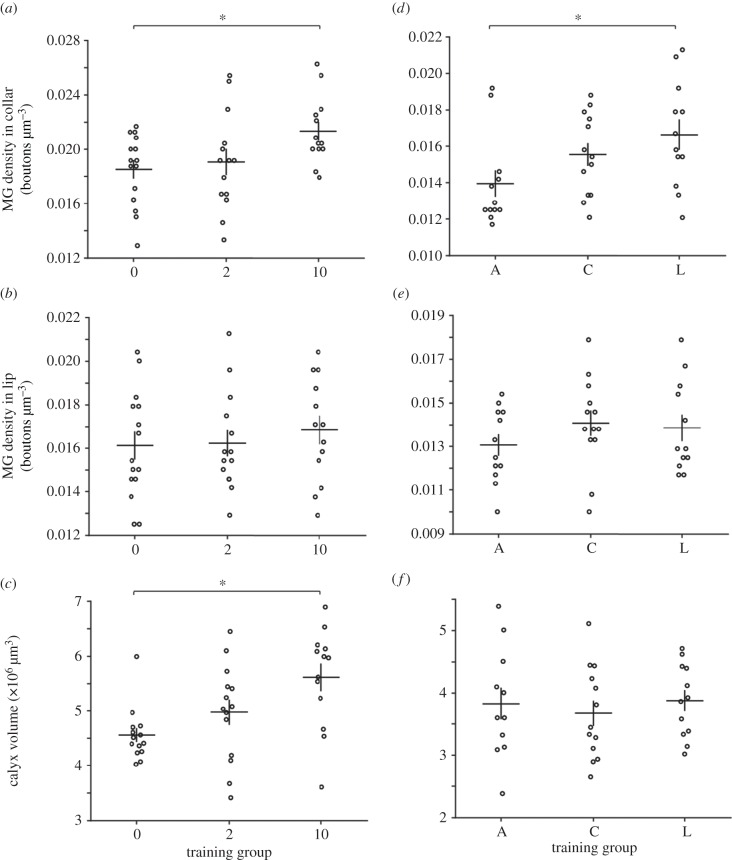
The effect of visual learning behaviour on microglomerular (MG) density. (*a–c*) 0, No-colour Learning (*n* = 15), 2, Two-colour Learning (*n* = 14); 10, 10-colour Learning (*n* = 13); experiment 3. Higher microglomerular density within the collar was found in the 10-colour Learning group compared to the No-colour Learning group (*a*; GLMM; *p* = 0.0156; electronic supplementary material, table S3). No differences in lip microglomerular density were found across groups (*b*; GLMM; electronic supplementary material, table S3). Calyx volume of the 10-colour Learning group was significantly higher than in the No-colour Learning group (*c*; GLMM; *p* = 0.0011; electronic supplementary material, table S3). (*d–f*) A, Activity Control group (*n* = 12); C, Colour Control group (*n* = 13); L, Learning group (*n* = 12); experiment 4. Microglomerular density in the collar of the Learning group was significantly higher than in the Activity Control group (*d*; GLMM; *p* = 0.0143; electronic supplementary material, table S4). There were no differences across these three groups for microglomerular density in the lip (*e*; GLMM; electronic supplementary material, table S4) or calyx volume (*f*; GLMM; electronic supplementary material, table S4). Asterisks indicate significant differences (*p* < 0.05). Horizontal bars indicate mean. Vertical bars indicate standard error of the mean.

To determine how visual learning affects microglomerular density, we exposed three groups of bees to varying levels of visual learning experiences (*n* = 42; No-colour Learning, Two-colour Learning and 10-colour Learning; experiment 3, Material and methods). The microglomerular density within the collar region of the mushroom bodies of bees in the 10-colour Learning group was higher compared with the No-colour Learning group (GLMM: *p* = 0.0156, figure 3*a*; electronic supplementary material, table S3). No differences in microglomerular density were found in the lip (GLMM, figure 3*b*; electronic supplementary material, table S3). However, the total calyx volume of the 10-colour Learning group bees was significantly different from No-colour Learning (GLMM: *p* = 0.0011, figure 3*c*; electronic supplementary material, table S3).

These differences in both microglomerular density and calyx volume, in the 10-colour Learning groups could be because of greater foraging activity during training or increased visual experience or visual learning. To determine to what degree, in our paradigm, learning induces changes in microglomerular density, we trained another three groups of bees in a similar paradigm (*n* = 37; Activity Control group, Colour Control group and Learning group; experiment 4; Material and methods; figure 1*b*). All three groups of bees experienced similar amounts of foraging activity during training. Both the Colour Control and Learning groups were exposed to similar colour information. Only the Learning group experienced colour learning. Microglomerular density in the mushroom body collar of the Learning group was significantly higher than the Activity Control group (GLMM: *p* = 0.0143, figure 3*d*; electronic supplementary material, table S4), suggesting that colour learning may have increased microglomerular density, rather than any changes that might be caused by just physical activity. However, there was no difference between microglomerular density in the mushroom body collar of the Learning group and the Colour Control group (GLMM, figure 3*d*; electronic supplementary material, table S4), suggesting that colour information may alone be responsible for the microglomerular density differences observed in our visual learning paradigm. Again, no differences in microglomerular density were found across these three groups in the lip region (GLMM, figure 3*e*; electronic supplementary material, table S4). Interestingly, the total calyx volume was not found to be different across groups (GLMM, figure 3*f*; electronic supplementary material, table S4), suggesting that foraging activity alone may be largely responsible for the calyces volumetric changes observed.

## 4. Discussion

### (a) General findings

We examined how the variation in learning and memory performance across individual bumblebees relates to microglomerular density in their brain. Individual bees that performed well in the retention test (experiment 1) and individual bees that learned quickly in training (experiment 2) had a relatively high microglomerular density in the visual input region of the mushroom body calyx, compared with bees that performed more poorly. This correlation was modality specific, as we found no correlation between microglomerular density in the lip region of the calyx and memory retention or learning speed. This was expected because the collar region of the calyx receives incoming visual information while the lip receives incoming olfactory information. Our results suggest that higher microglomerular density, which signifies more functional synapses [36,41,42], in the visual input region (calyx collar) of the brain may predispose bees to better visual learning and memory performance.

Can we determine to what extent learning contributed to memory performance through microglomerular changes? We found no difference between mean microglomerular density directly after learning in experiment 2 and mean microglomerular density after memory retention in experiment 1 (*t*-test; *t*_38_ = 0.2419, *p* = 0.8101). This obviously does not mean that learning does not cause changes in microglomerular density, especially given our experiments 3 and 4 results, but this might suggest that inter-individual differences in microglomerular density influence memory performance more than learning, in our paradigm. However, this type of comparison would really have to be done with bees from the same colony, which was not the case here. In addition, learning speed of individuals tested in experiment 1 and their memory retention did not correlate (GLMM; electronic supplementary material, table S5). This only suggests that learning probably causes varying degrees of changes in microglomerular density across individuals. The correlation (or lack of correlation) between these two measures is unhelpful for inferring the degree to which learning induced synaptic complex formation contributes to better memory performance. Higher microglomerular density however does seem to lead to faster learning speed and better memory retention.

We subsequently examined how microglomerular density and calyx volume differed depending on the numbers of colours learnt by bees (10 colours; two colours; no colours; experiment 3). Microglomerular density in the calyx collar, as well as the volume of the entire calyx, of bees that had learned 10 colours was significantly higher than in bees that had received no training or had learned two colours, indicating that long-term visual memory formation may result in greater microglomerular density in the collar and volumetric changes within the calyx in this specific task.

We then explored how visual learning, visual experience and foraging experience contributed to changes in neural architecture (experiment 4). Here, we examined how microglomerular density and calyx volume differed depending on whether bees learned to discriminate multiple colours (Learning), experienced the same colours without any learnt association (Colour), or experienced foraging without any flower-like colour exposure or learning (Activity). In the collar region, microglomerular density was significantly greater in the Learning group compared with the Activity Control group, while no difference was present between the Learning group and the Colour Control group. These results suggest that although colour learning may increase microglomerular density in the collar region of the mushroom body calyx, visual experience of colour (enrichment) alone may play a significant role in the microglomerular density differences seen in the Learning group.

We expect that the observed microglomerular density changes were owing to visual input from the projection neurons into the collar region of the mushroom bodies. However, we cannot rule out some contribution from feedback neurons from the mushroom body lobes [43,44], although the functional role of these neurons and the contribution of these mostly inhibitory neurons to synaptic plasticity is unknown.

### (b) Comparisons with previous works

Haenicke [45] using a classical conditioning paradigm, showed that odour response activity, measured via calcium imaging, of microglomeruli in the mushroom body calyx of the honeybee correlated with the expression level of individual short-term memory in the proboscis extension reflex. These results may provide a potential functional link to our finding that learning speed, a behavioural correlate of short-term memory, correlates with microglomerular density.

Sommerlandt *et al*. [29] found no difference in microglomerular density between honeybees trained to colours with differential conditioning or with absolute conditioning or ‘stimulus-naive’ bees. Differences in the age of bees, and prior experience before experiments, which were not controlled for in their study, might have obscured any genuine interactions of experimental treatment with microglomerular differences. In our study, the advantage of working with bumblebees was that both age and experience could more easily be controlled for, mainly because bumblebees, unlike honeybees, can easily and successfully live and forage in a laboratory hive box and arena. Sommerlandt *et al*. did find a correlation between the performance of honeybees that learned through differential conditioning and their microglomerular density in the mushroom body calyx. This weak negative correlation, as surmised by the authors, could be owing to variations in experience by the honeybees; pruning of neural connections with increased age and experience may correlate with better discrimination ability, or potentially less explorative behaviour [29]. Van Nest *et al*. [28] found no correlation between microglomerular density in the mushroom body calyces and honeybee performance on a two-colour visual discrimination task. In our study, we found a statistically strong positive correlation between performance on a visual discrimination task and microglomerular density in the mushroom body calyxes, and specifically in the visual input region (collar region). The reason for the difference in sign of correlation (with [28]) and significance (with [29]) may be owing to the differences in controls, as we controlled for both age and prior foraging experience. In addition, the 10-colour Learning task in our experiment was arguably more difficult than the traditional Two-colour Learning task used by these other studies. The increased difficulty of the visual task allowed for more variation in performance and is probably responsible for the correlation with microglomerular density we observed in bees.

Formation of long-term memory accompanying greater microglomerular density has been shown within the olfactory domain both in honeybees [25] and ants [26]. However, we show for the first time, to our knowledge, that changes in microglomerular density can be induced via acquisition of visual memory.

Structural plasticity can also manifest itself via more gross volumetric changes, especially in response to foraging experience [23,46,47]. However, Hourcade *et al*. [25] showed that long-term olfactory memory formation did not affect lip volume of honeybees after formation of odour memories. The apparent conflict of these results and ours may, again, be explained by the difference in difficulty of tasks used. Hourcade *et al*. trained honeybees to only one odour, and used restrained honeybees, which limited both physical activity and all incoming sensory information. We used free-flying bumblebees exposed to 10 different colours. In addition, others have shown that visual stimulation (exposure to sunlight or an artificial light source, for 45 min, five times, with 1 h 15 min inter-trial-intervals, for 3–4 days) induces volume increases in the collar of the mushroom body calyx in ants and honeybees [21,24]. It is possible that experiencing a greater number of different training stimuli during free-flight might be responsible for the volumetric changes we observed.

A plethora of studies have shown that increased number of environmental stimuli in which an animal interacts with (environmental enrichment) induces both structural and functional neural plasticity as well as improved learning and memory [48]. It may be that within our controlled environment of the laboratory, experience of 10 novel colours may represent an enriched environment able to induce significant structural reorganization in the visual regions of the brain.

In fact, the greater microglomerular density and calyx volume in the 10-colour Learning group could have resulted from a combination of foraging activity, colour stimulation or colour learning. Visual stimulation via light exposure is known to induce changes in microglomerular density [21,24,27]. But our work provides a strong correlation between microglomerular density and memory retention, a strong correlation between increased microglomerular density and faster learning, and evidence that increased experience with colours led to increased microglomerular density.

Intriguingly, there were no significant differences for the total calyx volume among Learning, Colour Control and Activity Control groups, in experiment 4, where we examined how colour learning, colour experience and foraging experience related to differences in microglomerular density and calyx volume. Our results indicate that foraging experience (or physical activity) may contribute to the total calyx volume changes in the 10-colour Learning group (experiment 3). Alternatively, stimulation beyond colour (spatial, direction, olfactory, tactile, etc.) may contribute to differences in whole calyx volume, which might have overshadowed any changes owing to learning or colour information. In fact, natural foraging activity has been shown to cause differences in the volume of mushroom bodies [47,49].

### (c) Ecological significance

Natural variations in learning speed and memory retention across bees may exist because of the need for colonies to explore new resources for food throughout the season as the floral patterns and food sources change over time, i.e. rather than making errors, some bees may be exploring alternative options [50,51]. Genetic differences across colonies and natural variations in microglomerular density across individual bees may predict bees' foraging performance, but they may also predict individual's exploratory behaviour or other behaviour yet untested. Future work should attempt to combine both measurements of learning performance and foraging behaviour.

In summary, our results show that microglomerular density in the visual input region (collar of calyx) of the bumblebee mushroom body positively correlates with memory retention and learning speed. This correlation does not imply causality, but is, to our knowledge, the first to suggest a link between synaptic complex density and individual bee learning performance (on a specific task). This is probably because our novel task design was more difficult for bees to learn, which led to a larger variation in individual performance and because in our laboratory setting, as opposed to bees' natural environment, we could control better for potential conflicting influences on microglomerular density. These new findings provide methods and a model animal for exploring how the brain encodes inter-individual differences in learning. We have shown that visual learning results in greater microglomerular density, and that exposure to colour information may play a significant role in experience-dependent changes in microglomerular density. Our findings add new insight into how visual learning and experience affect neural structure and suggest new avenues of research for studying the interplay between these phenomena.

## Supplementary Material

Supplementary Methodology

## Supplementary Material

Supplementary Figure S1: Specifications of colours used for all experiments

## Supplementary Material

Supplementary Tables S1-S6

## Supplementary Material

Supplementary Data for all experiments and analyses

## References

[1] Haier RJ 2011 Biological basis of intelligence. In The Cambridge handbook of intelligence *(eds RJ Sternberg, SB Kaufman)*, pp. 351–370. New York, NY: Cambridge University Press.

[2] Snell-Rood EC, Papaj DR, Gronenberg W 2009 Brain size: a global or induced cost of learning? Brain Behav. Evol. 73, 111–128. (10.1159/000213647) 19390176

[3] Benson-Amram S, Dantzer B, Stricker G, Swanson EM, Holekamp KE 2016 Brain size predicts problem-solving abilities in mammalian carnivores. Proc. Natl Acad. Sci. USA 113, 2532–2537. (10.1073/pnas.1505913113) 26811470 PMC4780594

[4] Healy SD, Rowe C 2007 A critique of comparative studies of brain size. Proc. R. Soc. B 274, 453–464. (10.1098/rspb.2006.3748) PMC1766390 17476764

[5] Bailey CH, Kandel ER 1993 Structural changes accompanying memory storage. Annu. Rev. Physiol. 55, 397–426. (10.1146/annurev.ph.55.030193.002145) 8466181

[6] Poo MM et al. 2016 What is memory? The present state of the engram. BMC Biol. 14, 40 (10.1186/s12915-016-0261-6) 27197636 PMC4874022

[7] Benfenati F 2007 Synaptic plasticity and the neurobiology of learning and memory. Acta Bio. Medica. Atenei. Parm. 78, 58–66. 17465325

[8] Fahrbach SE, Van Nest BN 2016 Synapsin-based approaches to brain plasticity in adult social insects. Curr. Opin. Insect Sci. 18, 1–8. (10.1016/j.cois.2016.08.009) 27939707

[9] Srinivasan MV 2010 Honey bees as a model for vision, perception, and cognition. Annu. Rev. Entomol. 55, 267–284. (10.1146/annurev.ento.010908.164537) 19728835

[10] Menzel R, Giurfa M 2001 Cognitive architecture of a mini-brain: the honeybee. Trends Cogn. Sci. 5, 62–71. (10.1016/S1364-6613(00)01601-6) 11166636

[11] Muller H, Chittka L 2012 Consistent interindividual differences in discrimination performance by bumblebees in colour, shape and odour learning tasks (Hymenoptera: Apidae: *Bombus terrestris*). Entomol. Gen. 34, 1 (10.1127/entom.gen/34/2012/1)

[12] Raine NE, Chittka L 2012 No trade-off between learning speed and associative flexibility in bumblebees: a reversal learning test with multiple colonies. PLoS ONE 7, e45096 (10.1371/journal.pone.0045096) 23028779 PMC3447877

[13] Heisenberg M 1998 What do the mushroom bodies do for the insect brain? An introduction. Learn. Mem. 5, 1–10. 10454369 PMC311238

[14] Heisenberg M 2003 Mushroom body memoir: from maps to models. Nat. Rev. Neurosci. 4, 266–275. (10.1038/nrn1074) 12671643

[15] Fahrbach SE 2006 Structure of the mushroom bodies of the insect brain. Annu. Rev. Entomol. 51, 209–232. (10.1146/annurev.ento.51.110104.150954) 16332210

[16] Ehmer B, Gronenberg W 2002 Segregation of visual input to the mushroom bodies in the honeybee (*Apis mellifera*). J. Comp. Neurol. 451, 362–373. (10.1002/cne.10355) 12210130

[17] Paulk AC, Gronenberg W 2008 Higher order visual input to the mushroom bodies in the bee, *Bombus impatiens*. Arthropod. Struct. Dev. 37, 443–458. (10.1016/j.asd.2008.03.002) 18635397 PMC2571118

[18] Gronenberg W 1986 Physiological and anatomical properties of optical input-fibres to the mushroom body in the bee brain. J. Insect Physiol. 32, 695 701–699 704. (10.1016/0022-1910(86)90111-3)

[19] Yasuyama K, Meinertzhagen IA, Schürmann F 2002 Synaptic organization of the mushroom body calyx in *Drosophila melanogaster*. J. Comp. Neurol. 445, 211–226. (10.1002/cne.10155) 11920702

[20] Groh C, Rossler W 2011 Comparison of microglomerular structures in the mushroom body calyx of neopteran insects. Arthropod. Struct. Dev. 40, 358–367. (10.1016/j.asd.2010.12.002) 21185946

[21] Stieb SM, Muenz TS, Wehner R, Rossler W 2010 Visual experience and age affect synaptic organization in the mushroom bodies of the desert ant *Cataglyphis fortis*. Dev. Neurobiol. 70, 408–423. (10.1002/dneu.20785) 20131320

[22] Krofczik S, Khojasteh U, de Ibarra NH, Menzel R 2008 Adaptation of microglomerular complexes in the honeybee mushroom body lip to manipulations of behavioral maturation and sensory experience. Dev. Neurobiol. 68, 1007–1017. (10.1002/dneu.20640) 18446779

[23] Groh C, Lu Z, Meinertzhagen IA, Rossler W 2012 Age-related plasticity in the synaptic ultrastructure of neurons in the mushroom body calyx of the adult honeybee *Apis mellifera*. J. Comp. Neurol. 520, 3509–3527. (10.1002/cne.23102) 22430260

[24] Stieb SM, Hellwig A, Wehner R, Rossler W 2012 Visual experience affects both behavioral and neuronal aspects in the individual life history of the desert ant *Cataglyphis fortis*. Dev. Neurobiol. 72, 729–742. (10.1002/dneu.20982) 21954136

[25] Hourcade B, Muenz TS, Sandoz JC, Rossler W, Devaud JM 2010 Long-term memory leads to synaptic reorganization in the mushroom bodies: a memory trace in the insect brain? J. Neurosci. 30, 6461–6465. (10.1523/JNEUROSCI.0841-10.2010) 20445072 PMC6632731

[26] Falibene A, Roces F, Rossler W 2015 Long-term avoidance memory formation is associated with a transient increase in mushroom body synaptic complexes in leaf-cutting ants. Front. Behav. Neurosci. 9, 84 (10.3389/fnbeh.2015.00084) 25904854 PMC4389540

[27] Scholl C, Wang Y, Krischke M, Mueller MJ, Amdam G V, Rossler W 2014 Light exposure leads to reorganization of microglomeruli in the mushroom bodies and influences juvenile hormone levels in the honeybee. Dev. Neurobiol. 74, 1141–1153. (10.1002/dneu.22195) 24890265

[28] Van Nest BN, Wagner AE, Marrs GS, Fahrbach SE 2017 Volume and density of microglomeruli in the honey bee mushroom bodies do not predict performance on a foraging task. Dev. Neurobiol. 77, 1057–1071. (10.1002/dneu.22492) 28245532

[29] Sommerlandt FMJ, Spaethe J, Rössler W, Dyer AG 2016 Does fine color discrimination learning in free-flying honeybees change mushroom- body calyx neuroarchitecture? PLoS ONE 11, e0164386 (10.1371/journal.pone.0164386) 27783640 PMC5081207

[30] Mares S, Ash L, Gronenberg W 2005 Brain allometry in bumblebee and honey bee workers. Brain. Behav. Evol. 66, 50–61. (10.1159/000085047) 15821348

[31] Riveros AJ, Gronenberg W 2010 Brain allometry and neural plasticity in the bumblebee *Bombus occidentalis*. Brain. Behav. Evol. 75, 138–148. (10.1159/000306506) 20516659 PMC2914411

[32] Hagen M, Dupont YL 2013 Inter-tegular span and head width as estimators of fresh and dry body mass in bumblebees (*Bombus* spp.). Insectes Soc. 60, 251–257. (10.1007/s00040-013-0290-x)

[33] Menzel R, Manz G, Menzel R, Greggers U 2001 Massed and spaced learning in honeybees: the role of CS, US, the intertrial interval, and the test interval. Learn. Mem. 8, 198–208. (10.1101/lm.40001) 11533223 PMC311375

[34] Nagerl UV, Kostinger G, Anderson JC, Martin KA, Bonhoeffer T 2007 Protracted synaptogenesis after activity-dependent spinogenesis in hippocampal neurons. J. Neurosci. 27, 8149–8156. (10.1523/JNEUROSCI.0511-07.2007) 17652605 PMC6672732

[35] Morimoto K, Sato K, Sato S, Yamada N, Hayabara T 1998 Time-dependent changes in rat hippocampal synapsin I mRNA expression during long-term potentiation. Brain Res. 783, 57–62. (10.1016/S0006-8993(97)01154-2) 9479047

[36] Hart AK, Fioravante D, Liu R-Y, Phares GA, Cleary LJ, Byrne JH 2011 Serotonin-mediated synapsin expression is necessary for long-term facilitation of the aplysia sensorimotor synapse. J. Neurosci. 31, 18 401–18 411. (10.1523/JNEUROSCI.2816-11.2011) PMC3407595 22171042

[37] Ott SR 2008 Confocal microscopy in large insect brains: zinc-formaldehyde fixation improves synapsin immunostaining and preservation of morphology in whole-mounts. J. Neurosci. Methods 172, 220–230. (10.1016/j.jneumeth.2008.04.031) 18585788

[38] Lunau K, Wacht S, Chittka L 1996 Colour choices of naive bumble bees and their implications for colour perception. J. Comp. Physiol. A 178, 477–489. (10.1007/BF00190178)

[39] Leadbeater E, Chittka L 2007 The dynamics of social learning in an insect model, the bumblebee (*Bombus terrestris*). Behav. Ecol. Sociobiol. 61, 1789–1796. (10.1007/s00265-007-0412-4)

[40] Ings TC, Raine NE, Chittka L 2009 A population comparison of the strength and persistence of innate colour preference and learning speed in the bumblebee *Bombus terrestris*. Behav. Ecol. Sociobiol. 63, 1207–1218. (10.1007/s00265-009-0731-8)

[41] Ferreira A, Chin LS, Li L, Lanier LM, Kosik KS, Greengard P 1998 Distinct roles of synapsin I and synapsin II during neuronal development. Mol. Med. 4, 22–28. 9513186 PMC2230269

[42] Sato K, Morimoto K, Suemaru S, Sato T, Yamada N 2000 Increased synapsin I immunoreactivity during long-term potentiation in rat hippocampus. Brain Res. 872, 219–222. (10.1016/S0006-8993(00)02460-4) 10924697

[43] Grünewald B 1999 Morphology of feedback neurons in the mushroom body of the honeybee, *Apis mellifera*. J. Comp. Neurol. 404, 114–126. (10.1002/(SICI)1096-9861(19990201)404:1%3C114::AID-CNE9%3E3.0.CO;2-#) 9886029

[44] Strausfeld NJ 2002 Organization of the honey bee mushroom body: representation of the calyx within the vertical and gamma lobes. J. Comp. Neurol. 450, 4–33. (10.1002/cne.10285) 12124764

[45] Haenicke J 2015 Modeling insect inspired mechanisms of neural and behavioral plasticity. Dissertation, Freie Universität, Berlin, Germany.

[46] Ismail N, Robinson GE, Fahrbach SE 2006 Stimulation of muscarinic receptors mimics experience-dependent plasticity in the honey bee brain. Proc. Natl Acad. Sci. USA 103, 207–211. (10.1073/pnas.0508318102) 16373504 PMC1324993

[47] Maleszka J, Barron AB, Helliwell PG, Maleszka R 2009 Effect of age, behaviour and social environment on honey bee brain plasticity. J. Comp. Physiol. A 195, 733–740. (10.1007/s00359-009-0449-0) 19434412

[48] Van Praag H, Kempermann G, Gage FH 2000 Neural consequences of environmental enrichment. Nat. Rev. Neurosci. 1, 191–198. (10.1038/35044558) 11257907

[49] Farris SM, Robinson GE, Fahrbach SE 2001 Experience-and age-related outgrowth of intrinsic neurons in the mushroom bodies of the adult worker honeybee. J. Neurosci. 21, 6395–6404. 11487663 10.1523/JNEUROSCI.21-16-06395.2001 PMC6763189

[50] Raine NE, Chittka L 2008 The correlation of learning speed and natural foraging success in bumble-bees. Proc. R. Soc. B 275, 803–808. (10.1098/rspb.2007.1652) PMC2596909 18198141

[51] Woodgate JL, Makinson JC, Lim KS, Reynolds AM, Chittka L 2016 Life-long radar tracking of bumblebees. PLoS ONE 11, e0160333 (10.1371/journal.pone.0160333) 27490662 PMC4973990

