# Supplementary material for: A possible structural correlate of learning performance on a colour discrimination task in the brain of the bumblebee
Source: Proc Biol Sci. 2017 Oct 4;284(1864):20171323. doi: 10.1098/rspb.2017.1323 (PMC5647297; doi:10.1098/rspb.2017.1323)
Supplement: Supplementary Methodology [file rspb20171323supp1.pdf]

A possible structural correlate of learning performance on a colour discrimination task in the brain of the bumblebee

Li Li, HaDi MaBouDi, Michaela Egertová, Maurice R. Elphick, Lars Chittka, Clint J. Perry*
School of Biological and Chemical Sciences, Queen Mary University of London, London E1 4NS, UK

Corresponding Author: Clint J. Perry, School of Biological and Chemical Sciences, Queen Mary University of London, London E1 4NS, UK, clint.perry@qmul.ac.uk

Supplementary Material: Supplementary Methodology

S1. Illumination of arena and colour stimuli

The illumination in the room came from fluorescent lighting (TL-D 58W/840, Philips, Eindhoven, The Netherlands) fitted with high-frequency ballasts (Tridonic PC 2/70 T8 Pro, Dornbirn, Austria) to generate lighting above the bee flicker fusion frequency. The spectral range was 400-700nm; nonetheless there was some (small) stimulation of UV receptors since their sensitivity in *Bombus terrestris* extends slightly above 400nm.

Bees were number-tagged under red light to ensure colour information for bees was kept at a minimum. Bees were able to enter the front section of the nest box to defecate and to remove debris from the nest area. This front section was partially illuminated through a Perspex lid, however, this area contained no flower-like patterns or colours and no food was accessible from this area so no colour-reward associations could be learned. Illumination was controlled with a 12 h day-night cycle (8:00 am - 8:00 pm).

The colour loci of the flowers were calculated using the actual irradiance spectrum in the flight arena. Spectral reflectance of the chips was measured under standardised conditions (as described in [1]) – by definition, spectral reflectance is given relative to a white standard and is therefore independent of illumination.

Chip colours were chosen with relatively even distribution across the RGB spectrum. Chip spectral reflectance functions were measured in the laboratory using a spectrophotometer (Avantes AvaSpec-2048) with deuterium halogen source (AvaLight-DHS). The spectral

reflectance and colour information of the 10 coloured chips are shown in figure S1a and 1b. The colour loci of stimuli were calculated in a hexagon colour space [2] (figure S1c) considering the published spectral sensitivity functions of bumblebee photoreceptors [3].

S2. Quantification of microglomeruli in the mushroom body calyces

We established a methodology for immunolabelling of presynaptic terminals in whole-mount brains that enabled identification of microglomeruli, employing an antibody to the synaptic vesicle-associated protein synapsin I. Synapsin is a presynaptic vesicle-associated protein shown to regulate new synapse formation [4] and associated with long-term memory formation [5–7], although its function within insect microglomeruli is undefined. Our method combined the procedures from two previous studies [8,9]. Immediately after collection, bees were anesthetized with CO_2 by holding them a few centimeters above dry ice for 5 seconds. Up to five bees could be tested sequentially and collected each day of experiments. Once anesthetized, each bee was kept at -20°C for approximately 10 min, enough time for the bee to be completely anesthetized throughout dissection. Once the last of the tested bees had been placed at -20°C, the first bee was removed from -20°, the head was then removed and head width was measured with Vernier calipers. To dissect the bee brain, each bee's head was kept on ice and a rectangular window was cut in the head capsule to expose the brain. The semi-dissected heads were immediately immersed in ice-cold 4% formaldehyde and kept overnight at 4°C. For each bee, the fixed head capsule was washed in phosphate buffer saline (PBS) twice, the tracheae, glands and thin membrane surrounding the brain were removed and then the brain was dissected from the head capsule, all under PBS. After washes in 1% dimethyl sulfoxide (DMSO) in PBS (PBS/DMSO) (3×10 min) and in 0.2% Triton X-100 (Tx) in PBS/DMSO (PBS/DMSO/TX) (3×10 min), brains were permeabilized in 80% methanol/20% DMSO for two hours and then rehydrated through a methanol series (100% methanol, 1 hour;

90%, 70%, 50%, 30%, and 0% methanol in 0.1 M Tris buffer, pH 7.4, 10 min each). Prior to incubation in primary antibodies, brains were blocked in 5% normal goat serum (NGS; G9023-10ML, Sigma-Aldrich Company Ltd., Dorset, UK) in PBS/DMSO at 4°C overnight. For synapsin immunolabelling, brains were incubated in a monoclonal mouse antibody against the Drosophila synaptic vesicle-associated protein synapsin I (SYNORF1, kindly provided by E. Buchner, University of Würzburg, Germany), diluted 1:10 in PBS/DMSO with 5% NGS for three days at 4°C. After several washes in PBS/DMSO/TX and then PBS/DMSO (one day), brains were incubated in Alexa Fluor 594–conjugated goat anti-mouse secondary antibody (115-585-062, Jackson ImmunoResearch Laboratories Inc., West Grove, PA) (1:800) in 2.5% normal goat serum in PBS/DMSO for 2.5 days at 4°C. Brains were then washed in PBS/DMSO/TX (5×10 min) and PBS/DMSO (one day) and cleared in an ascending glycerol series (25%, 50%, 75% in PBS) until the brain sank to the bottom of the tube. Finally, the brains were stored in an anti-fade mounting medium (1:9 PBS:glycerol (ACS grade 99-100% purity) with 0.1 part 20% n-propyl gallate (Sigma P3130) added dropwise with rapid stirring). To ensure that no cell shrinkage or fractionation occurred during preparation we repeated the same methods but stained brains with DAPI. Good nuclear morphology in the DAPI-stained image indicated no fractionation and very limited shrinkage (data not shown). The whole brains were scanned using a laser-scanning confocal microscope (Leica SP5). For microglomeruli measurement, z-stacks were created by taking optical sections at 0.5 µm intervals with a x63 oil immersion objective at a resolution of 1,024 × 1,024 pixels. For calyx volume measurements, z-stacks were created by taking optical sections at 5 µm intervals with a x20 oil immersion objective at a resolution of 1,024 × 1,024 pixels. Digital images were processed using 3D reconstruction software Imaris 7.6 (Bitplane AG, Zürich, Switzerland). Spheroidal structures of ~2.5µm were clearly visible at high magnification. These microglomeruli represent distinct synaptic complexes in the calyx neuropil, each comprising a central bouton

from projection neuron axons surrounded by many KC dendritic spines and processes from other extrinsic neurons [10,11]. Synapsin, which is associated with synaptic vesicles, stained the central bouton of the microglomeruli. Five cuboid volumes (7.8 µm × 7.8 µm × 7.8 µm) were manually selected in the lip or collar regions and the microglomeruli were automatically counted in the defined regions according to the diameter and staining intensity with background subtraction (figure 1*c*-*g*). The microglomeruli counts for both collar and lip regions of the calyces were determined by the Imaris 7.6 spot function. The spot function is created by framing the cuboidal volumes within each specific region layer by layer through the 3D structure. The diameter range of the microglomeruli was defined as being between 2.0 and 3.0 µm, set by measurement of the microglomeruli through the Imaris function. Setting the diameter lower than this range introduced background noise, and setting the diameter higher caused miscalculation by overlapping parts. Results were visually confirmed to ensure that all defined microglomeruli in this range were counted within each sampled section. The average microglomerular density for each bee was then calculated by dividing the average number of microglomeruli found in each cuboid by the volume of a cuboid, $474.552 \mu m^2$. The five regions were dispersed uniformly throughout the lip or dense collar region of one lateral calyx of each bee. We chose, as others have, to sample from only the dense collar region because this outer region of the collar receives visual information from the optic lobe medulla [12] and contains a more uniform distribution of microglomeruli (e.g. [13]); synapsin-positive microglomeruli are densely packed and homogenously distributed here [8]. Our methods limit our ability to determine whether the microglomerular densities come from regions that receive input from the dorsal or ventral medulla. Improved methods could allow for the determination from what region of the medulla the change in microglomerular density received input visual information, allowing for a more mechanistic understanding of the inputs and outputs. The lip region is an oval-shaped structure on the top of each calyx branch and the collar region displays areas of

dense and sparse staining. Note that measurements of entire calyx volume were used in our study because it was not possible to determine the boundary between collar and lip regions in each brain and therefore volume estimates of these regions would not be reliable. To ensure each cube was positioned distinctly within either the lip or the collar region, any area within 5 μm of these boundaries were avoided. Cubes were placed 1 μm away from the outer edge of the lip and collar. Counting and analyses were conducted blindly, as files were code-named by one individual and analysed by a different individual. The number of microglomeruli in the lip and collar regions were averaged separately and the resulting means were used for later analyses.

S3. Statistical analyses

For memory retention assessment (Experiment 1), the proportion of landings on rewarding chips in the retention test for each bee was calculated. A landing was defined as any time the bee was positioned on top of a chip and not flying for any amount of time. For learning speed (improvement on learning performance over trips) assessment (Experiment 2), a learning curve was obtained by fitting a first-order exponential decay function to the number of errors in each ten landings for each bee [14]. An error was defined as any time the bee landed on top of an incorrect chip. The number of errors a bee made per number of choices were plotted and an exponential decay function with the equation $y = y_0 + Ae^{-x/t}$ was fitted to the data, where x is the number of flower choices the bee made since it entered the arena, and y is the number of errors. The saturation performance level (y_0) is the errors made by a bee after finishing the learning process, i.e. when reaching a performance plateau (final asymptotic value of the y value). A is the curve amplitude (the maximum height of the curve above y_0). The decay constant (t) is a measure of learning speed: high values of t correspond to slow learning, whereas lower t values indicate faster learning. These t values were used for subsequent

analysis. Generalized linear mixed models (GLMM) were used to examine the effects of predictors on memory retention. Collar microglomerular density, lip microglomerular density and calyx volume were fixed factors, and age, head width, total number of landings and colony were random factors, for the predictor of memory retention. The same was done for learning speed except that colony and age were not included, since only one colony was used in Experiment 2 and age was not included since all bees were 12 days old. For Experiments 3 and 4, GLMMs were used to examine the effects of the different groups on microglomerular density. Colony was not considered since only one colony was used for each of Experiments 3 and 4. Bee age and head width were random factors. No correlation was found between colony (n = 3, in Experiment 1), age (10-16 days), head width (4.2-5.1mm) or number of landings (54-124) and any of the predictors in any of the experiments.

References

1. Chittka L, Kevan PG. 2005 Flower colour as advertisement. In *Practical Pollination Biology* (eds A Dafni, PG Kevan, BC Husband), pp. 157–196. Enviroquest Ltd., Cambridge, ON, Canada.
2. Chittka L. 1992 The colour hexagon: a chromaticity diagram based on photoreceptor excitations as a generalized representation of colour opponency. *J. Comp. Physiol. A* **170**, 533–543.
3. Skorupski P, Döring TF, Chittka L. 2007 Photoreceptor spectral sensitivity in island and mainland populations of the bumblebee, Bombus terrestris. *J. Comp. Physiol. A Neuroethol. Sensory, Neural, Behav. Physiol.* **193**, 485–494. (doi:10.1007/s00359-006-0206-6)
4. Ferreira A, Chin LS, Li L, Lanier LM, Kosik KS, Greengard P. 1998 Distinct roles of synapsin I and synapsin II during neuronal development. *Mol. Med.* **4**, 22–28.
5. Hart AK, Fioravante D, Liu R-Y, Phares GA, Cleary LJ, Byrne JH. 2011 Serotonin-Mediated Synapsin Expression Is Necessary for Long-Term Facilitation of the Aplysia Sensorimotor Synapse. *J. Neurosci.* **31**, 18401–18411. (doi:10.1523/JNEUROSCI.2816-11.2011)
6. Sato K, Morimoto K, Suemaru S, Sato T, Yamada N. 2000 Increased synapsin I immunoreactivity during long-term potentiation in rat hippocampus. *Brain Res.* **872**, 219–222. (doi:10.1016/S0006-8993(00)02460-4)
7. Morimoto K, Sato K, Sato S, Yamada N, Hayabara T. 1998 Time-dependent changes in rat hippocampal synapsin I mRNA expression during long-term potentiation. *Brain Res.* **783**, 57–62.
8. Groh C, Lu Z, Meinertzhagen IA, Rossler W. 2012 Age-related plasticity in the synaptic ultrastructure of neurons in the mushroom body calyx of the adult honeybee

Apis mellifera. *J Comp Neurol* **520**, 3509–3527. (doi:10.1002/cne.23102)

9. Ott SR. 2008 Confocal microscopy in large insect brains: zinc-formaldehyde fixation improves synapsin immunostaining and preservation of morphology in whole-mounts. *J Neurosci Methods* **172**, 220–230. (doi:10.1016/j.jneumeth.2008.04.031)
10. Yasuyama K, Meinertzhagen IA, Schürmann F. 2002 Synaptic organization of the mushroom body calyx in Drosophila melanogaster. *J. Comp. Neurol.* **445**, 211–226.
11. Groh C, Rossler W. 2011 Comparison of microglomerular structures in the mushroom body calyx of neopteran insects. *Arthropod Struct Dev* **40**, 358–367. (doi:10.1016/j.asd.2010.12.002)
12. Ehmer B, Gronenberg W. 2002 Segregation of visual input to the mushroom bodies in the honeybee (Apis mellifera). *J. Comp. Neurol.* **451**, 362–373.
13. Stieb SM, Muenz TS, Wehner R, Rossler W. 2010 Visual experience and age affect synaptic organization in the mushroom bodies of the desert ant Cataglyphis fortis. *Dev Neurobiol* **70**, 408–423. (doi:10.1002/dneu.20785)
14. Raine NE, Chittka L. 2008 The correlation of learning speed and natural foraging success in bumble-bees. *Proc Biol Sci* **275**, 803–808. (doi:10.1098/rspb.2007.1652)

a

Human Colour	+	+	-	+ *	-	+ *	-	+	-	+	-
RGB values	242,242,242	255,1,1	255,152,1	243,255,1	152,255,1	1,255,61	1,255,213	1,182,255	61,1,255	182,1,255	255,1,213
Hexagon Loci (x,y)	0.1418, 0.0308	-0.0100, 0.0688	0.2145, -0.0759	0.3324, -0.1029	0.2668, -0.0729	0.2157, -0.0497	0.3001, 0.0729	0.2874, 0.1961	0.1177, 0.2648	0.1338, 0.2476	0.1658, 0.2522

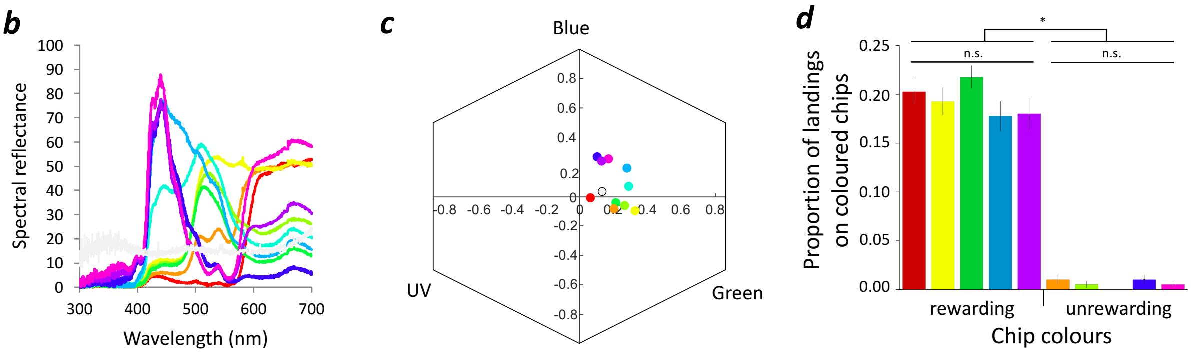

Figure S1. Colours used for all experiments. (*a*) Human visual depiction of each of the colours used in experiments, with RGB values and bee vision hexagon loci. +/- symbols indicate rewarding (+) and unrewarding (-) chips during training. Asterisks indicate yellow and green chips used for 2-colour Learning group in Experiment Experiment 3. (*b*) Spectral reflectance plot of each of the colours used. (*c*) Loci of chip colours in bee colour space, describing the range of colours a bee can see given their three photoreceptors sensitive to Blue, Green and UV light. Dots indicate each of the chip colours used in the experiments and are shown with human depicted colours. The closer to the center the dot, the greyer the colour appears to the bee, and the closer to the edge, the brighter the colour appears. The closer the dots are together the more similar they look to a bee. (*d*) Histogram of landings among rewarding colours during training. During the last 10 landings of training, bees landed more on all rewarding colours than any unrewarding colours (GLMM: $p < 0.0001$; table S6), but there was no difference amongst rewarding colours and no difference amongst unrewarding colours.

Table S1. Summary of generalized linear mixed models examining memory retention factors in relation to microglomerular density (Experiment 1).

Dependent variable	Fixed factors	df	Estimate	SE	*F*	*P*
Memory retention	Intercept	1	43.55	11.01	15.65	0.0052
	**MG density in collar**	1	2699.40	545.63	24.48	**$3.862e^{-5}$**
	MG density in lip	1	67.11	854.12	0.01	0.9380
	Total calyx volume	1	$-1.90e^{-6}$	$2.10e^{-6}$	0.82	0.3735

The dependent variable was the percentage correct choices during the memory retention test. The MG density in the collar, MG density in the lip, and the total calyx volume were included as fixed factors. Age, head width, number of landings and colony (N = 3) were included as a random factors. The significant terms are highlighted in bold.

Table S2. Summary of generalized linear mixed models examining learning speed factors in relation to microglomerular density (Experiment 2).

Dependent variable	Fixed factors	df	Estimate	SE	*F*	*P*
Learning speed	Intercept	1	14.65	3.5138	17.39	0.0059
	**MG density in collar**	1	-560.47	139.08	16.24	**0.0069**
	MG density in lip	1	-185.00	205.70	0.8089	0.4031
	Total calyx volume	1	$-1.89e^{-7}$	$6.11e^{-7}$	0.0959	0.7672

The dependent variable was the t-value calculated for learning speed during training. The MG density in the collar, MG density in the lip, and the total calyx volume were included as fixed factors. Age and colony were not included as random factors because all bees were 12 days old and from the same colony. Head width and number of landings were included as random factors. The significant terms are highlighted in bold.

Table S3. Summary of generalized linear mixed models examining training condition factors in relation to microglomerular density (Collar or Lip) or Calyx Volume (Experiment 3).

Dependent variable	Fixed factors	df	Estimate	SE	*F*	*P*
Collar MG density	Intercept	1	0.0185	0.0008	595.64	0.0000
	Two colour learning	2	0.0005	0.0011	0.25	0.6185
	**Ten colour learning**	2	0.0028	0.0011	6.40	**0.0156**

The dependent variable was the MG density in the collar. The training conditions were included as fixed factors. Age and headwidth were included as random factors. The reference condition was the clear chip training (no colour learning). The significant terms are highlighted in bold.

Dependent variable	Fixed factors	df	Estimate	SE	*F*	*P*
Lip MG density	Intercept	1	0.0157	$0.09e^{-3}$	277.55	0.0000
	Training condition	1	0.0004	$0.45e^{-6}$	0.6258	0.4336

The dependent variable was the MG density in the lip.

Dependent variable	Fixed factors	df	Estimate	SE	*F*	*P*
Calyx volume	Intercept	1	$4.56e^{6}$	$2.07e^{5}$	480.59	0.0000
	Two colour learning	2	$0.42e^{6}$	$2.94e^{5}$	2.04	0.1614
	**Ten colour learning**	2	$1.06e^{6}$	$3.00e^{5}$	12.52	**0.0011**

The dependent variable was the volume of the calyx. The significant terms are highlighted in bold.

Table S4. Summary of generalized linear mixed models examining training condition factors in relation to microglomerular density (Collar or Lip) or Calyx Volume (Experiment 4).

Dependent variable	Fixed factors	df	Estimate	SE	*F*	*P*
Collar MG density	Intercept	1	0.0166	0.0007	506.72	0.0000
	**Activity control**	2	-0.0027	0.0010	6.67	**0.0143**
	Colour control	2	-0.0011	0.0010	1.16	0.2895

The dependent variable was the MG density in the collar. The training conditions were included as fixed factors. Age and headwidth were included as random factors. The reference condition was the Learning condition. The significant terms are highlighted in bold.

Dependent variable	Fixed factors	df	Estimate	SE	*F*	*P*
Lip MG density	Intercept	1	0.0129	$8.69e^{-4}$	220.13	0.0000
	Training condition	1	0.0004	$4.03e^{-3}$	0.97	0.3326

The dependent variable was the MG density in the lip.

Dependent variable	Fixed factors	df	Estimate	SE	*F*	*P*
Calyx volume	Intercept	1	$3.74e^{6}$	$3.36e^{5}$	123.72	0.0000
	Training Condition	2	$0.26e^{6}$	$1.54e^{5}$	0.03	0.8658

The dependent variable was the calyx volume.

Table S5. Summary of generalized linear mixed models examining memory retention factors in relation to learning speed (Experiment 1).

Dependent variable	Fixed factors	df	Estimate	SE	*F*	*P*
Memory retention	Intercept	1	87.48	3.6531	573.36	$3.4373e^{-20}$
	Learning speed	1	-1.4536	1.2654	1.3197	0.2604

The dependent variable was the percentage correct choices during the memory retention test. The learning speed was included as a fixed factor. Age and headwidth and colony (N = 3) were included as random factors. The significant terms are highlighted in bold.

Table S6. Summary of generalized linear mixed models examining landings in relation to colour of chip and rewarding value (rewarding/unrewarding).

Dependent variable	Fixed factors	df	Estimate	SE	*F*	*P*
Landings	Intercept	1	0.4140	0.0339	149.18	$2.4508e^{-29}$
	Colour	1	-0.0115	0.0072	2.5326	0.1123
	**Value**	1	-0.2020	0.0283	50.7371	$\mathbf{5.0154e^{-12}}$
	Colour*Value	1	0.0046	0.0046	1.4482	0.2295

The dependent variable was the proportion of landings on each colour during the last ten trails of training. The chip colour and value (rewarding/unrewarding) of each chip were included as a fixed factors. Individual bee was included as a random factor. The significant terms are highlighted in bold.
