# Supplementary material for: A possible structural correlate of learning performance on a colour discrimination task in the brain of the bumblebee
Source: Proc Biol Sci. 2017 Oct 4;284(1864):20171323. doi: 10.1098/rspb.2017.1323 (PMC5647297; doi:10.1098/rspb.2017.1323)
Supplement: Supplementary Figure S1: Specifications of colours used for all experiments [file rspb20171323supp2.pdf]

A possible structural correlate of learning performance on a colour discrimination task in the brain of the bumblebee

Li Li, HaDi MaBouDi, Michaela Egertová, Maurice R. Elphick, Lars Chittka, Clint J. Perry*
School of Biological and Chemical Sciences, Queen Mary University of London, London E1 4NS, UK

Corresponding Author: Clint J. Perry, School of Biological and Chemical Sciences, Queen Mary University of London, London E1 4NS, UK, clint.perry@qmul.ac.uk

Supplementary Material: Figure S1

a

Human Colour	+	+	-	+ *	-	+ *	-	+	-	+	-
RGB values	242,242,242	255,1,1	255,152,1	243,255,1	152,255,1	1,255,61	1,255,213	1,182,255	61,1,255	182,1,255	255,1,213
Hexagon Loci (x,y)	0.1418, 0.0308	-0.0100, 0.0688	0.2145, -0.0759	0.3324, -0.1029	0.2668, -0.0729	0.2157, -0.0497	0.3001, 0.0729	0.2874, 0.1961	0.1177, 0.2648	0.1338, 0.2476	0.1658, 0.2522

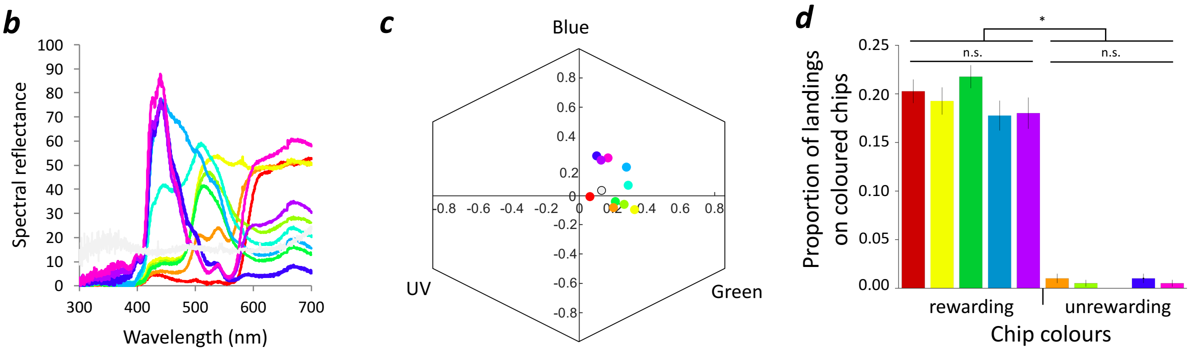

Figure S1. Specifications of colours used for all experiments. (*a*) Human visual depiction of each of the colours used in experiments, with RGB values and bee vision hexagon loci. +/- symbols indicate rewarding (+) and unrewarding (-) chips during training. Asterisks indicate yellow and green chips used for 2-colour Learning group in Experiment Experiment 3. (*b*) Spectral reflectance plot of each of the colours used. (*c*) Loci of chip colours in bee colour space, describing the range of colours a bee can see given their three photoreceptors sensitive to Blue, Green and UV light. Dots indicate each of the chip colours used in the experiments and are shown with human depicted colours. The closer to the center the dot, the greyer the colour appears to the bee, and the closer to the edge, the brighter the colour appears. The closer

the dots are together the more similar they look to a bee. (*d*) Histogram of landings among rewarding colours during training. During the last 10 landings of training, bees landed more on all rewarding colours than any unrewarding colours (GLMM: $p < 0.0001$; table S6), but there was no difference amongst rewarding colours and no difference amongst unrewarding colours.
