# Supplementary material for: A possible structural correlate of learning performance on a colour discrimination task in the brain of the bumblebee
Source: Proc Biol Sci. 2017 Oct 4;284(1864):20171323. doi: 10.1098/rspb.2017.1323 (PMC5647297; doi:10.1098/rspb.2017.1323)
Supplement: Supplementary Tables S1-S6 [file rspb20171323supp3.pdf]

A possible structural correlate of learning performance on a colour discrimination task in the brain of the bumblebee

Li Li, HaDi MaBouDi, Michaela Egertová, Maurice R. Elphick, Lars Chittka, Clint J. Perry*
School of Biological and Chemical Sciences, Queen Mary University of London, London E1 4NS, UK

Corresponding Author: Clint J. Perry, School of Biological and Chemical Sciences, Queen Mary University of London, London E1 4NS, UK, clint.perry@qmul.ac.uk

Supplementary Material: Tables S1-S6

Table S1. Summary of generalized linear mixed models examining memory retention factors in relation to microglomerular density (Experiment 1).

Dependent variable	Fixed factors	df	Estimate	SE	*F*	*P*
Memory retention	Intercept	1	43.55	11.01	15.65	0.0052
	**MG density in collar**	1	2699.40	545.63	24.48	**$3.862e^{-5}$**
	MG density in lip	1	67.11	854.12	0.01	0.9380
	Total calyx volume	1	$-1.90e^{-6}$	$2.10e^{-6}$	0.82	0.3735

The dependent variable was the percentage correct choices during the memory retention test. The MG density in the collar, MG density in the lip, and the total calyx volume were included as fixed factors. Age, head width, number of landings and colony (N = 3) were included as a random factors. The significant terms are highlighted in bold.

Table S2. Summary of generalized linear mixed models examining learning speed factors in relation to microglomerular density (Experiment 2).

Dependent variable	Fixed factors	df	Estimate	SE	*F*	*P*
Learning speed	Intercept	1	14.65	3.5138	17.39	0.0059
	**MG density in collar**	1	-560.47	139.08	16.24	**0.0069**
	MG density in lip	1	-185.00	205.70	0.8089	0.4031
	Total calyx volume	1	$-1.89e^{-7}$	$6.11e^{-7}$	0.0959	0.7672

The dependent variable was the t-value calculated for learning speed during training. The MG density in the collar, MG density in the lip, and the total calyx volume were included as fixed factors. Age and colony were not included as random factors because all bees were 12 days old and from the same colony. Head width and number of landings were included as random factors. The significant terms are highlighted in bold.

Table S3. Summary of generalized linear mixed models examining training condition factors in relation to microglomerular density (Collar or Lip) or Calyx Volume (Experiment 3).

Dependent variable	Fixed factors	df	Estimate	SE	*F*	*P*
Collar MG density	Intercept	1	0.0185	0.0008	595.64	0.0000
	Two colour learning	2	0.0005	0.0011	0.25	0.6185
	**Ten colour learning**	2	0.0028	0.0011	6.40	**0.0156**

The dependent variable was the MG density in the collar. The training conditions were included as fixed factors. Age and headwidth were included as random factors. The reference condition was the clear chip training (no colour learning). The significant terms are highlighted in bold.

Lip MG density	Intercept	1	0.0157	$0.09e^{-3}$	277.55	0.0000
	Training condition	1	0.0004	$0.45e^{-6}$	0.6258	0.4336

The dependent variable was the MG density in the lip.

Calyx volume	Intercept	1	$4.56e^{6}$	$2.07e^{5}$	480.59	0.0000
	Two colour learning	2	$0.42e^{6}$	$2.94e^{5}$	2.04	0.1614
	**Ten colour learning**	2	$1.06e^{6}$	$3.00e^{5}$	12.52	**0.0011**

The dependent variable was the volume of the calyx. The significant terms are highlighted in bold.

Table S4. Summary of generalized linear mixed models examining training condition factors in relation to microglomerular density (Collar or Lip) or Calyx Volume (Experiment 4).

Dependent variable	Fixed factors	df	Estimate	SE	*F*	*P*
Collar MG density	Intercept	1	0.0166	0.0007	506.72	0.0000
	**Activity control**	2	-0.0027	0.0010	6.67	**0.0143**
	Colour control	2	-0.0011	0.0010	1.16	0.2895

The dependent variable was the MG density in the collar. The training conditions were included as fixed factors. Age and headwidth were included as random factors. The reference condition was the Learning condition. The significant terms are highlighted in bold.

Lip MG density	Intercept	1	0.0129	$8.69e^{-4}$	220.13	0.0000
	Training condition	1	0.0004	$4.03e^{-3}$	0.97	0.3326

The dependent variable was the MG density in the lip.

Calyx volume	Intercept	1	$3.74e^{6}$	$3.36e^{5}$	123.72	0.0000
	Training Condition	2	$0.26e^{6}$	$1.54e^{5}$	0.03	0.8658

The dependent variable was the calyx volume.

Table S5. Summary of generalized linear mixed models examining memory retention factors in relation to learning speed (Experiment 1).

Dependent variable	Fixed factors	df	Estimate	SE	*F*	*P*
Memory retention	Intercept	1	87.48	3.6531	573.36	$3.4373e^{-20}$
	Learning speed	1	-1.4536	1.2654	1.3197	0.2604

The dependent variable was the percentage correct choices during the memory retention test. The learning speed was included as a fixed factor. Age and headwidth and colony (N = 3) were included as random factors. The significant terms are highlighted in bold.

Table S6. Summary of generalized linear mixed models examining landings in relation to colour of chip and rewarding value (rewarding/unrewarding).

Dependent variable	Fixed factors	df	Estimate	SE	*F*	*P*
Landings	Intercept	1	0.4140	0.0339	149.18	$2.4508e^{-29}$
	Colour	1	-0.0115	0.0072	2.5326	0.1123
	**Value**	1	-0.2020	0.0283	50.7371	**$5.0154e^{-12}$**
	Colour*Value	1	0.0046	0.0046	1.4482	0.2295

The dependent variable was the proportion of landings on each colour during the last ten trails of training. The chip colour and value (rewarding/unrewarding) of each chip were included as a fixed factors. Individual bee was included as a random factor. The significant terms are highlighted in bold.
