# Supplementary material for: A possible structural correlate of learning performance on a colour discrimination task in the brain of the bumblebee
Source: Proc Biol Sci. 2017 Oct 4;284(1864):20171323. doi: 10.1098/rspb.2017.1323 (PMC5647297; doi:10.1098/rspb.2017.1323)
Supplement: Supplementary Data for all experiments and analyses [file rspb20171323supp4.pdf]

Experiment 1

performance	lip density	collar density	total volume	colony	leaning speed	age	head width	number landings
95	0.0158	0.0163	4980000	1	1.25			109
86	0.0133	0.0154	2483600	1	0.5593			54
81	0.0150	0.0146	3400000	1	7.3638			101
81	0.0133	0.0138	4100000	1	2.9621			89
67	0.0121	0.0138	3160000	1	0.1071			77
65	0.0171	0.0121	4340000	1	0.1029			83
100	0.0196	0.0221	5970000	2	0.1237	14		60
89	0.0171	0.0254	5620000	2	0.8127	11		94
86	0.0171	0.0183	5990000	2	0.1228	14	4.4	84
96	0.0129	0.0204	3612000	2	0.495	12	5	87
91	0.0188	0.0200	6140000	2	1.4438	12		102
100	0.0179	0.0225	6210000	2	3.5633	10		114
81	0.0158	0.0209	5233000	2	2.0431	10	4.6	99
75	0.0142	0.0179	5540000	2	2.2143	14	4.9	115
73	0.0196	0.0204	6900000	2	1.6741	14	4.8	93
100	0.0163	0.0263	6090000	2	0.7211	15	4.6	98
100	0.0138	0.0229	4660000	2	3.0723	15	4.8	94
100	0.0154	0.0200	4530000	2	2.825	14	4.6	111
95	0.0204	0.0200	6530000	2	0.1327	13	5	108
50	0.0142	0.0121	3020000	3	5.9424	15		104
95	0.0129	0.0192	3581000	3	2.0802	14	4.4	90
88	0.0125	0.0179	3330000	3	0.9138	12	4.6	114
79	0.0167	0.0179	3929000	3	2.419	15	4.4	82
89	0.0117	0.0209	4710000	3	2.4836	10	4.5	91
85	0.0125	0.0138	3140000	3	1.6159	10	4.5	86
88	0.0129	0.0154	4620000	3	3.5259	11	4.8	124
100	0.0179	0.0213	3380000	3	0.6179	11	4.8	121
70	0.0158	0.0154	3870000	3	0.9579	16	4.5	95
70	0.0117	0.0167	4120000	3	2.0714	12	4.3	80
85	0.0121	0.0158	4430000	3	0.1397	13	4.8	115

Experiment 2

learning speed	lip density	collar density	total volume	age	head width	number landings
7.8385	0.0129	0.0138	3630000	12	4.7	119
4.2579	0.0175	0.0138	5550000	12	4.8	107
2.9471	0.0146	0.0167	4201000	12	4.4	78
0.9988	0.0154	0.0175	3911000	12	4.4	74
2.6378	0.0121	0.0213	4779000	12		98
1.566	0.0133	0.0213	4570000	12	4.7	87
2.3415	0.0121	0.0179	4960000	12	4.4	81
1.3965	0.0108	0.0200	5060000	12	4.4	75
0.9897	0.0121	0.0204	3467000	12	4.3	60
0.1347	0.0179	0.0238	6180000	12	4.7	61

Last Ten Landings

1	2	3	4	5	6	7	8	9	10
2	5	3	1	2	3	4	1	3	2
4	3	5	7	2	1	3	5	1	4
2	1	5	2	3	2	1	9	5	3
3	2	5	1	5	1	4	3	2	2
5	3	2	3	1	2	4	3	1	4
7	3	1	1	3	9	4	2	1	4
2	3	5	1	1	4	1	4	1	4
1	5	3	4	5	3	2	2	5	1
5	1	4	2	4	5	1	2	3	2
3	2	6	2	1	2	5	4	2	2
4	5	5	9	2	3	1	2	5	3
3	2	5	1	3	4	4	5	3	1
5	3	2	3	5	3	4	1	3	2
3	5	2	1	2	4	1	5	1	3
4	4	4	2	1	5	2	5	3	4
4	5	2	1	5	1	5	4	5	3
6	5	5	4	4	5	3	2	1	3
5	2	1	3	4	2	1	3	1	4
5	3	1	2	1	2	4	5	3	1
2	1	2	3	5	1	2	3	1	3
3	10	1	4	3	5	3	5	4	4
5	9	2	4	4	3	1	3	1	3
1	3	5	3	2	3	5	2	4	5
1	2	3	1	2	5	3	4	3	1
3	3	1	5	1	2	4	3	2	1
4	5	1	1	2	4	3	3	2	5
5	4	3	4	1	6	3	2	2	3
4	1	3	2	4	4	3	4	2	4
3	5	3	1	5	1	5	4	2	1
5	3	2	4	1	3	1	5	4	5
5	2	4	5	10	1	5	3	5	5
1	2	5	3	1	4	5	2	4	3
1	1	4	4	3	5	4	2	3	2
6	4	2	2	1	3	2	4	1	5
3	1	2	3	1	4	2	1	3	5
4	2	3	5	1	2	4	5	3	1
2	3	4	1	2	4	5	3	1	2
2	5	3	4	3	1	4	2	4	1
3	1	5	4	2	1	5	3	4	3
1	3	4	3	2	1	5	4	5	2

Numbers refer to Figure S1a from red to magenta

Experiment 3

group	lip density	collar density	total volume	age	head width
3	0.0196	0.0221	5970000	14	
3	0.0171	0.0254	5620000	11	
3	0.0171	0.0183	5990000	14	4.4
3	0.0129	0.0204	3612000	12	5
3	0.0188	0.0200	6140000	12	
3	0.0179	0.0225	6210000	10	
3	0.0158	0.0209	5233000	10	4.6
3	0.0142	0.0179	5540000	14	4.9
3	0.0196	0.0204	6900000	14	4.8
3	0.0163	0.0263	6090000	15	4.6
3	0.0138	0.0229	4660000	15	4.8
3	0.0154	0.0200	4530000	14	4.6
3	0.0204	0.0200	6530000	13	5
2	0.0154	0.0204	4840000	14	
2	0.0175	0.0192	6095000	11	
2	0.0154	0.0167	5410000	12	5.1
2	0.0183	0.0192	5450000	12	4.6
2	0.0142	0.0179	3677000	10	4.2
2	0.0146	0.0167	5250000	10	5
2	0.0150	0.0229	6450000	14	4.8
2	0.0158	0.0254	5728300	13	4.9
2	0.0167	0.0192	3419000	16	4.7
2	0.0146	0.0163	4090000	16	4.6
2	0.0129	0.0146	4180000	15	4.6
2	0.0196	0.0200	5080000	15	4.6
2	0.0158	0.0133	5040000	13	4.6
2	0.0213	0.0250	4970000	13	4.6
1	0.0204	0.0150	4720000	14	
1	0.0179	0.0217	4400000	14	
1	0.0146	0.0200	4360000	11	
1	0.0171	0.0192	4260000	13	
1	0.0183	0.0200	4559900	12	4.4
1	0.0138	0.0188	4230000	10	4.7
1	0.0150	0.0154	4602000	10	5
1	0.0179	0.0209	4970000	14	4.8
1	0.0150	0.0188	5990000	13	4.8
1	0.0200	0.0213	4020000	13	4.9
1	0.0154	0.0171	4066000	17	4.7
1	0.0125	0.0192	4700000	15	4.9
1	0.0167	0.0213	4413000	15	4.5
1	0.0146	0.0163	4510000	13	4.7
1	0.0125	0.0129		16	4.5

1 = no colour learning (clear chips) 2 = 2 colour learning 3 = 10 colour learning

Experiment 4

group	lip density	collar density	total volume	age	head width
3	0.0142	0.0121	3020000	15	4.2
3	0.0154	0.0133	3581000	13	4.5
3	0.0129	0.0192	3330000	14	4.4
3	0.0125	0.0179	3929000	12	4.6
3	0.0167	0.0179	4710000	15	4.4
3	0.0117	0.0209	3140000	10	4.5
3	0.0125	0.0138	4620000	10	4.5
3	0.0129	0.0154	3380000	11	4.8
3	0.0179	0.0213	3870000	11	4.8
3	0.0158	0.0154	4120000	16	4.5
3	0.0117	0.0167	4430000	12	4.3
3	0.0121	0.0158	4400000	13	4.8
2	0.0146	0.015	3450000	13	4.5
2	0.0133	0.0146	2940000	11	4.2
2	0.0163	0.0129	5110000	15	4.5
2	0.015	0.0133	4440000	14	4.4
2	0.0146	0.0179	4450000	12	4.7
2	0.0158	0.0188	2890000	12	4.5
2	0.0179	0.0133	4080000	10	4.6
2	0.0108	0.0183	4231300	15	4.4
2	0.0138	0.0158	3810000	11	4.9
2	0.0138	0.0175	3110000	11	5
2	0.0133	0.0171	3330000	12	4.8
2	0.0138	0.0154	2645000	12	4.7
2	0.01	0.0121	3280000	13	4.8
1	0.0146	0.0146	2380000	11	4.4
1	0.0117	0.0117	4510000	15	4.5
1	0.0121	0.0138	3090000	14	4.7
1	0.0133	0.0142	3600000	12	4.8
1	0.0146	0.0121	5387400	12	4.8
1	0.0113	0.0125	4010000	10	4.4
1	0.0154	0.0192	3130000	11	4.8
1	0.0142	0.0125	3600000	15	4.8
1	0.01	0.0125	5010000	16	4.4
1	0.0125	0.0125	3320000	12	4.8
1	0.0121	0.0129	4100000	12	4.8
1	0.015	0.0188		13	4.8

1 = activity control (clear)

2 = colour control (clear + 10 colour not landed on)

3 = colour learning (5 colour + 5 colour -)
